# Olive Leaf Extract Attenuates Obesity in High-Fat Diet-Fed Mice by Modulating the Expression of Molecules Involved in Adipogenesis and Thermogenesis

**DOI:** 10.1155/2014/971890

**Published:** 2014-01-28

**Authors:** Ying Shen, Su Jin Song, Narae Keum, Taesun Park

**Affiliations:** Department of Food and Nutrition, Brain Korea 21 PLUS Project, Yonsei University, 50 Yonsei-ro, Seodaemun-gu, Seoul 120-749, Republic of Korea

## Abstract

The present study aimed to investigate whether olive leaf extract (OLE) prevents high-fat diet (HFD)-induced obesity in mice and to explore the underlying mechanisms. Mice were randomly divided into groups that received a chow diet (CD), HFD, or 0.15% OLE-supplemented diet (OLD) for 8 weeks. OLD-fed mice showed significantly reduced body weight gain, visceral fat-pad weights, and plasma lipid levels as compared with HFD-fed mice. OLE significantly reversed the HFD-induced upregulation of WNT10b- and galanin-mediated signaling molecules and key adipogenic genes (PPAR**γ**, C/EBP**α**, CD36, FAS, and leptin) in the epididymal adipose tissue of HFD-fed mice. Furthermore, the HFD-induced downregulation of thermogenic genes involved in uncoupled respiration (SIRT1, PGC1**α**, and UCP1) and mitochondrial biogenesis (TFAM, NRF-1, and COX2) was also significantly reversed by OLE. These results suggest that OLE exerts beneficial effects against obesity by regulating the expression of genes involved in adipogenesis and thermogenesis in the visceral adipose tissue of HFD-fed mice.

## 1. Introduction

Obesity is an energy balance disorder in which energy intake exceeds energy expenditure, resulting in excessive accumulation of white adipose tissue (WAT) [1]. Adipogenesis is the process by which the mesenchymal precursor cells differentiate into adipocytes [2]. Research over the past decade has established the Wnt/*β*-catenin signaling pathway as an important regulator of adipogenesis [3]. WNT10b expression inhibits adipogenesis by suppressing the expression of transcription factors, proliferator-activated receptor gamma (PPAR*γ*), and CCAAT/enhancer-binding protein alpha (C/EBP*α*) [4], thereby inhibiting WAT development *in vivo*. Galanin has also been reported to play an essential role in regulating adipogenesis in animal models [5]. HFD-induced adipogenesis in the visceral adipose tissue of mice can be activated through the expression of galanin and its receptors together with the expression of the downstream molecules such as Ras, c-Raf, protein kinase C delta (PKC*δ*), and extracellular signal-regulated kinases (ERKs) that stimulate the activation of PPAR*γ* and C/EBP*α* [5]. Thus, therapeutic agents that increase WNT10b expression and decrease galanin expression may suppress the expression of adipogenic molecules, thereby inhibiting adipogenesis [6].

Unlike WAT, brown adipose tissue (BAT) is an essential component of energy expenditure as it dissipates energy in the form of heat [7], a process known as thermogenesis. Adipose mitochondria play a pivotal role in adaptive thermogenesis, the key adipose-specific metabolic pathway that is regulated by peroxisome proliferator-activated receptor *γ* coactivator-1alpha (PGC-1*α*) [8]. This pathway oxidizes lipids and dissipates heat energy as a result of the uncoupling of the mitochondrial electron transport chain due to ATP production by uncoupling protein 1 (UCP1). Thermogenesis is elevated in the mitochondria-rich BAT, and it is also observed in WAT, which contains brown-like cells [9, 10]. Thermogenesis has been demonstrated to be modified by macronutrient content in the diet, dietary carbohydrate, and fat type [11, 12]. Phytochemicals like resveratrol [13] and epigallocatechin gallate [14] have been reported to increase energy expenditure and thermogenesis in animals and humans. Therefore, it is possible that novel therapeutic agents activating or elevating the expression of PGC-1*α* and UCP1 would have crucial impacts on increasing the energy expenditure to ultimately prevent obesity.

Olive (*Olea europaea*) leaf has been widely used in traditional remedies as well as in human diet as an extract, in herbal tea and in the powder form in the European and Mediterranean countries [15]. Olive leaf extract (OLE) is marketed as a natural nutraceutical with wide-ranging health benefits. In recent years, research has mainly focused on the effects of extracts from olive leaves related to the prevention of hypertension [16], atherosclerosis [17], cancer [18], diabetes [19], and cardiovascular diseases [20, 21]. Olive leaves contain several different compounds collectively termed as olive biophenols, which impart the therapeutic properties. The most abundant biophenol is oleuropein, followed by other biophenols such as verbascoside, luteolin, rutin, catechin, and hydroxytyrosol in lower quantities [22]. Our previous study demonstrated that the addition of oleuropein to HFD decreased body weight gain and improved the lipid profiles in the plasma of mice [23]. Although several studies have investigated the biochemical roles of olive leaves and olive leaves preparations, their protective effect against diet-induced obesity has never been reported. Therefore, the objective of this study was to investigate the weight-reducing effect of a well-defined OLE by supplementation in HFD-induced mice and to investigate the underlying mechanisms of this effect, with a particular focus on the expression of molecules involved in adipogenesis and thermogenesis.

## 2. Materials and Method

### 2.1. Materials

Dry OLE was obtained from Frutarom Switzerland Ltd. (Wädenswil, Switzerland) under the trade name of Benolea. The extract was manufactured from the dried leaves of *Olea europaea* L., applying an ethanol (80%, m/m) extraction procedure. After a patented filtration process (EFLA Hyperpure), the crude extract was dried. Finally, 15% (m/m) Acaciae Gummi Ph. Eur was added as a carrier together with <0.5% (m/m) of silica colloidalis anhydrica Ph. Eur. The extract is characterized by a DER of 3–7 = 1 (5 = 1). Characteristic component is 16–24% (m/m) oleuropein. Sibutramine was purchased from Sigma-Aldrich (MA, USA).

### 2.2. High-Performance Liquid Chromatography (HPLC) Analysis of OLE

HPLC analysis was conducted using the Nova-Pak C18 column (150 mm × 3.9 mm, 60 A, Waters Associates, Harrow, UK) at 25°C at a flow rate of 1 mL/min by using a gradient mobile phase (water : methanol in 600 : 400 ratio) as the initial condition of the chromatography. The sample injection volume used was 10 *μ*L. The absorption signal of oleuropein was examined at 233 nm by using the UV detector (Agilent Technologies, DE, USA).

### 2.3. Animal and Diets

Five-week-old male C57BL/6N mice (Orient Bio, Gyeonggi-do, Korea) were housed in a pathogen-free facility at 21 ± 2.0°C, with 50 ± 5% relative humidity and a 12 h light/dark cycle; the mice were provided access to rodent chow and tap water *ad libitum* for 1 week. Thereafter, the mice were divided into the following 4 groups matched for body weight (*n* = 8 in each group): the chow diet (CD), high-fat diet (HFD), 0.15% (w/w) OLE-supplemented diet (OLD), and 0.01% (w/w) sibutramine-supplemented diet (SD) groups. The HFD consisted of 200 g fat/kg body weight (170 g of lard and 30 g of corn oil) and 1% (w/w) cholesterol. The OLD was identical to the HFD and contained 0.15% OLE. The SD was also identical to the HFD, except that it was supplemented with 0.01% sibutramine. The composition of the experimental diets including total energy (kcal/kg diet) is shown in Table 1. All the diets were provided in the form of pellets for 8 weeks.

The food intake was monitored daily, and body weights were recorded on a weekly basis. At the end of the experimental period, the mice were anesthetized with diethyl ether after they were fasted for 16 h. Blood samples were drawn from the inferior vena cava into an ethylene-diamine-tetra-acetic acid (EDTA)-coated tube, and the plasma samples were obtained by centrifuging the blood at 4000 ×g for 15 min at 4°C. The epididymal, retroperitoneal, mesenteric, and perirenal fat-pads were dissected, removed, weighed, and immediately snap-frozen in liquid nitrogen and stored at −80°C until further use. All animal experiments were performed in accordance with the Korea Food and Drug Administration (KFDA) guidelines. All the experimental protocols were reviewed and approved by the Institutional Animal Care and Use Committee (IACUC) of the Yonsei Laboratory Animal Research Center (YLARC).

### 2.4. Histological Analysis

The epididymal fat-pads were fixed in 10% buffered formalin and embedded in paraffin blocks. The paraffin blocks were sectioned and stained with hematoxylin-eosin (H&E). The sectional areas of the epididymal fat-pads were analyzed to quantify the size of the fat droplets.

### 2.5. Biochemical Analysis

Plasma concentrations of total cholesterol, triglyceride, HDL cholesterol, free fatty acid, and glucose were enzymatically determined using commercial kits (Bio-Clinical System, Gyeonggi-do, South Korea). Plasma levels of LDL + VLDL cholesterol were calculated by subtracting the HDL cholesterol level from the total cholesterol level. Plasma level of leptin was measured using a commercially available mouse enzyme-linked immunosorbent assay (ELISA) kit (Millipore, MA, USA).

### 2.6. RNA Extraction and Semiquantitative Reverse Transcriptase-Polymerase Chain Reaction (RT-PCR)

Total RNA was isolated from the epididymal adipose tissue of each mouse by using TRIzol (Invitrogen, CA, USA) and was reverse-transcribed using the superscript II kit (Invitrogen) according to the manufacturer's instructions. The GenBank accession numbers of the relevant templates and the forward (F) and reverse (R) primer sequences are shown in Table 2. The PCR was programmed as follows: 10 min at 94°C, 30–35 cycles of 94°C for 30 s, 55°C for 30 s, 72°C for 1 min, and 10 min incubation at 72°C. Then, 5 *μ*L of each PCR product was mixed with 1 *μ*L of 6-fold concentrated loading buffer; this solution was run on a 2% agarose gel containing ethidium bromide. The band intensities were quantified using the Quantity One Analysis Software (Bio-Rad, CA, USA). The mRNA levels were normalized to that of the glyceraldehyde-3-phosphate dehydrogenase (GAPDH) transcript.

### 2.7. Western Blotting

The epididymal adipose tissue samples obtained from each mouse were homogenized in an extraction buffer containing 100 mM Tris-HCl (pH 7.4), 5 mM EDTA, 50 mM NaCl, 50 mM sodium pyrophosphate, 50 mM NaF, 100 mM orthovanadate, 1% Triton X-100, 1 mM phenylmethanesulfonyl fluoride, 2 *μ*g/mL aprotinin, 1 *μ*g/mL pepstatin A, and 1 *μ*g/mL leupeptin. The homogenates were centrifuged at 1,300 ×g for 20 min at 4°C. The protein concentrations of the tissue extracts were measured by Bradford assay (Bio-Rad). The protein samples were separated by 8% sodium dodecyl sulfate (SDS)-polyacrylamide gel electrophoresis (PAGE) and then electrophoretically transferred to nitrocellulose membranes (Amersham, Buckinghamshire, UK). The nitrocellulose membranes were incubated overnight with primary antibodies (diluted 1 : 1,000) at 4°C. Antibodies to the following proteins were commercially obtained from the indicated sources: *β*-catenin and *β*-actin from Santa Cruz Biotechnology (Santa Cruz, CA, USA) and extracellular signal-related kinase (ERK), phospho-ERK (Thr202/Tyr204), cyclic AMP-responsive element-binding protein (CREB), and phospho-CREB (Ser133) from Cell Signaling Technology (MA, USA). The membranes were incubated with the corresponding secondary antibodies and the immunoreactive signals were detected using a chemiluminescent detection system (Amersham), followed by quantification using the Quantity One Analysis Software (Bio-Rad).

### 2.8. Statistical Analysis

The results on body weight gain, plasma biochemistries, and adipocyte diameter are presented as the mean ± standard error of mean (SEM) of 8 mice in each group. The RT-PCR and western blotting data are shown as the means ± SEM of three independent experiments (*n* = 2 or 3 per experiment) for each group, cumulatively including 8 mice. The statistical significance of difference was determined using one-way analysis of variance, followed by Duncan's multiple-range tests. All statistical analyses were performed with SPSS 21.0 software. *P* ≤ 0.05 was considered statistically significant.

## 3. Results

### 3.1. HPLC Analysis of OLE

**Figure 1:**
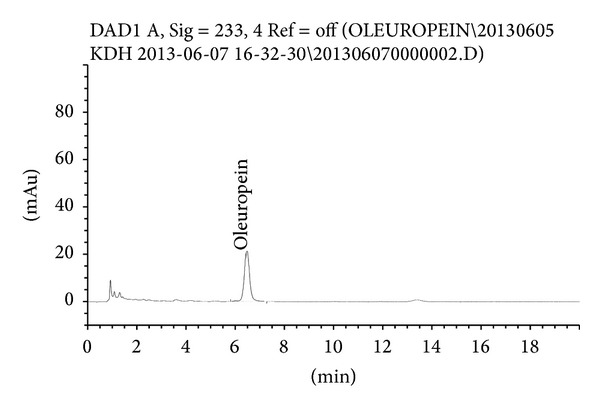
High-performance liquid chromatography (HPLC) chromatogram of the olive leaf extract (OLE). The peak was assigned based on the isolation of oleuropein.

HPLC analysis revealed that the OLE used in the present study, Benolea, contained 16% oleuropein (Figure 1).

### 3.2. Effects of OLE on Body and Visceral Fat-Pad Weights

**Figure 2:**
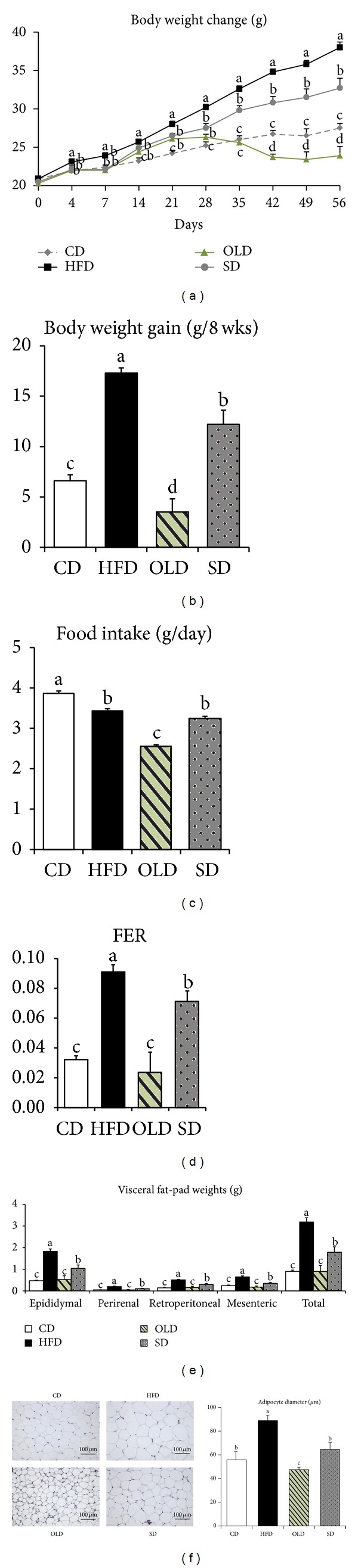
Effects of OLE on body weight gain, food efficiency ratio (FER), and visceral fat-pad weights of HFD-fed mice. Mice were fed CD, HFD, OLD, or SD for 8 weeks. Changes in the (a) body weight, (b) body weight gain, (c) food intake, (d) FER, and (e) visceral fat-pad weights. (f) Histological analysis of the epididymal adipose tissue from each group was conducted to quantify the size of adipocyte. (Left) Representative pictures of hematoxylin-eosin (H&E)-stained fat cells from the mice epididymal adipose tissue (×100). (Right) Densitometric analysis of the average adipocyte diameter in the epididymal adipose tissue of mice. Values are presented as means ± SEM (*n* = 8). Mean values indicated with different letters indicate statistical significance (*P* < 0.05); FER = Body weight gain for experimental period (g)/food intake for experimental period (g).

HFD-fed mice showed significant increases in final body weight (38%, *P* < 0.05) and cumulative body weight gain (162%, *P* < 0.05) as compared with CD-fed mice. OLD-fed mice showed a 37% reduction in final body weight and an 80% decrease in body weight gain as compared with HFD-fed mice (Figures 2(a) and 2(b)). The food intake was also significantly lower in OLD-fed mice (−26%, *P* < 0.05) than in HFD-fed mice during the 8-week feeding period. The food efficiency ratio (FER) (−74%, *P* < 0.05) of OLD-fed mice was significantly lower than that of HFD-fed mice (Figures 2(c) and 2(d)). The cumulative caloric intake over 8 weeks was 26% less in OLD-fed mice than in HFD-fed mice (660 versus 887 kcal). The OLD group also showed significantly lower values for total visceral (epididymal, mesenteric, perirenal, and retroperitoneal) fat-pad weights (−72%, *P* < 0.05) than those observed in the HFD group. Histological sections from the epididymal adipose tissue of HFD-fed mice presented a greater adipocyte diameter as compared with CD-fed mice; this change in the adipocyte size was significantly reversed by OLE supplementation (−47%, *P* < 0.05) (Figures 2(e) and 2(f)). OLE had greater effects on lowering body weight, food intake, total visceral fat-pad weights, and adipocyte size than did sibutramine (Figures 2(a)–2(f)).

### 3.3. Effects of OLE on Plasma Biochemistries

**Figure 3:**
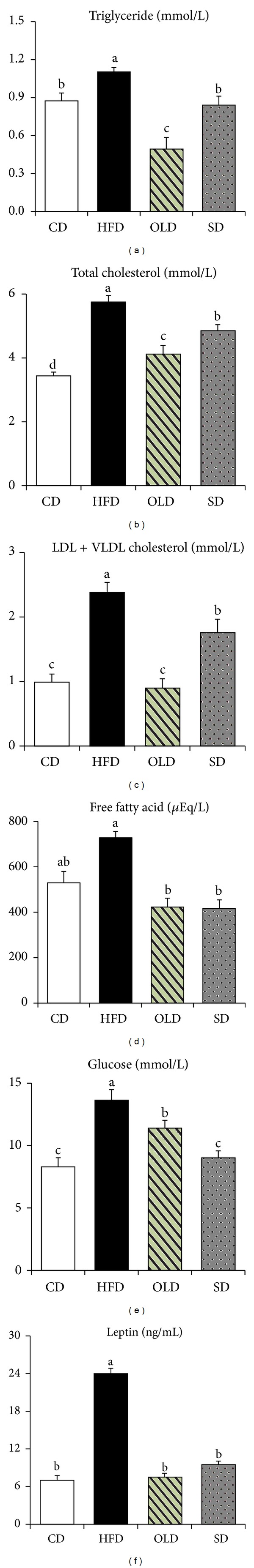
Effects of OLE on plasma levels of lipids, glucose, and leptin in HFD-fed mice. (a) Triglyceride, (b) total cholesterol, (c) LDL + VLDL cholesterol, (d) free fatty acid, (e) glucose, and (f) leptin. Bars represent the mean ± SEM (*n* = 8). Mean values indicated with different letters indicate statistical significance (*P* < 0.05).

OLE supplementation resulted in significantly lower concentrations of triglyceride (−55%, *P* < 0.05), total cholesterol (−28%, *P* < 0.05), LDL + VLDL cholesterol (−62%, *P* < 0.05), FFA (−42%, *P* < 0.05), glucose (−17%, *P* < 0.05), and leptin (−69%, *P* < 0.05) in the plasma of HFD-fed mice (Figures 3(a)–3(f)). The effect of OLE on reducing the plasma levels of triglyceride, total cholesterol, and LDL + VLDL cholesterol was significantly greater than that of sibutramine (Figures 3(a)–3(c)). OLE significantly reduced the plasma FFA and leptin levels, similar to sibutramine (Figures 3(d) and 3(f)); however, OLE was less effective in reducing the plasma glucose level than was sibutramine (Figure 3(e)).

### 3.4. Effects of OLE on the Expression of Molecules That Regulate Adipogenesis

**Figure 4:**
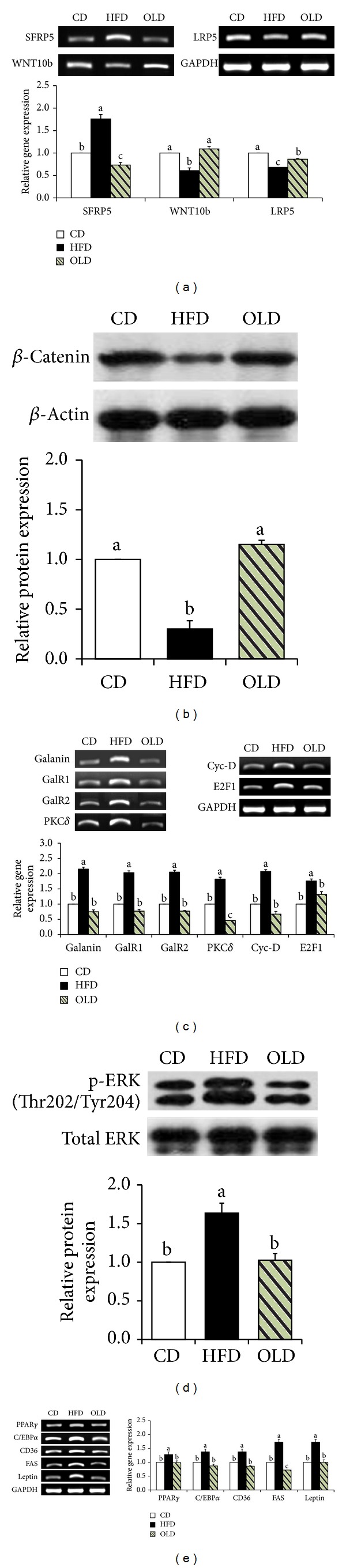
Effects of OLE on genes regulating adipogenesis in the epididymal adipose tissue of HFD-fed mice. (a) Gene expression of SFRP5, WNT10b, and LRP5, (b) protein levels of *β*-catenin, (c) mRNA expression of genes involved in the galanin-mediated signaling pathway, (d) protein levels of p-ERK and total ERK, (e) expression of PPAR*γ*2, C/EBP*α*, and their target genes. Reverse transcriptase-polymerase chain reaction (RT-PCR) data represent the relative density normalized to that of glyceraldehyde-3-phosphate dehydrogenase (GAPDH). Protein levels were normalized to the *β*-actin level. Data represent the results of three independent experiments (*n* = 2 or 3 per experiment); *P* < 0.05 indicates statistical significance. Values are the means ± SEM, *n* = 8 for each group.

We investigated whether OLE affected HFD-induced activation of adipogenesis in the epididymal adipose tissue of mice. In the present study, we found that OLE significantly upregulated the expression of wingless-type MMTV integration site family, member 10b (WNT10b) (79%, *P* < 0.05), and low-density lipoprotein receptor-related protein 5 (LRP5) (26%, *P* < 0.05) and downregulated the expression of secreted frizzled-related protein 5 (SFRP5) (−59%, *P* < 0.05) in the epididymal adipose tissue of HFD-fed mice (Figure 4(a)). Western blotting of the epididymal adipose tissue of mice revealed a significantly higher protein level of *β*-catenin (300%, *P* < 0.05) in OLD-fed mice relative to HFD-fed mice (Figure 4(b)). Furthermore, compared with HFD-fed mice, OLD-fed mice showed significantly lower expression of galanin (−65%, *P* < 0.05), galanin receptor 1 (GalR1) (−62%, *P* < 0.05), galanin receptor 2 (GalR2) (−62%, *P* < 0.05), PKC*δ* (−74%, *P* < 0.05), cyclin D (Cyc-D) (−68%, *P* < 0.05), and E2F1 (−25%, *P* < 0.05) in the epididymal adipose tissue of mice (Figure 4(c)). OLE supplementation resulted in decreased protein levels of phosphorylated ERK (Thr202/Tyr204) (−38%, *P* < 0.05) in HFD-fed mice (Figure 4(d)). The mRNA levels of C/EBP*α*, PPAR*γ*, and its target genes (CD36, FAS, and leptin) were significantly downregulated in OLD-fed mice as compared with those in HFD-fed mice (Figure 4(e)).

### 3.5. Effects of OLE on the Expression of Molecules Involved in Thermogenesis

**Figure 5:**
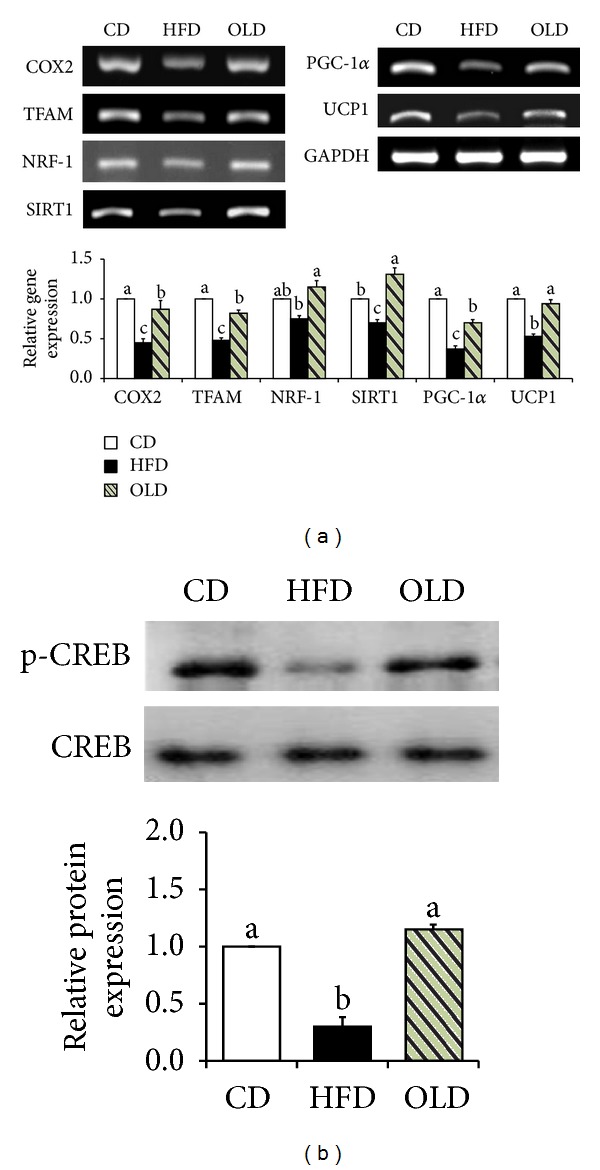
Effects of OLE on the expression of genes involved in thermogenesis in the epididymal adipose tissue of HFD-fed mice. (a) The expression of genes involved in uncoupled respiration and mitochondrial biogenesis was determined by RT-PCR and normalized to that of GAPDH; (b) protein levels of p-CREB and total CREB were determined by western blotting and normalized to *β*-actin protein levels. Data represent the results of three independent experiments (*n* = 2 or 3 per experiment); *P* < 0.05 indicates statistical significance. Values are the means ± SEM, *n* = 8 for each group.

We next assessed whether OLE can induce thermogenesis in the epididymal adipose tissue of mice. The mRNA levels of mitochondrial protein cytochrome C oxidase subunit 2 (COX2) (93%, *P* < 0.05), mitochondrial transcription factor A (TFAM) (71%, *P* < 0.05), nuclear respiratory factor-1 (NRF-1) (53%, *P* < 0.05), sirtuin 1 (SIRT1) (87%, *P* < 0.05), PGC-1*α* (89%, *P* < 0.05), and UCP1 (77%, *P* < 0.05) were significantly elevated in OLD-fed mice than in HFD-fed mice (Figure 5(a)). OLE supplementation increased the protein level of phosphorylated CREB (450%, *P* < 0.05) in the epididymal adipose tissue of HFD-fed mice (Figure 5(b)).

## 4. Discussion

OLE is best known for its blood pressure-lowering effect in the global market of functional food industry. In 2008, OLE, under the trade name Benolea, was approved by KFDA as a health functional food that exerts blood pressure-lowering effect at a daily recommended dose of 500–1000 mg. In addition, a clinical trial conducted by Perrinjaquet-Moccetti et al. confirmed that no adverse event was observed at a daily given dose of 1000 mg [21]. The concentration of OLE (0.15%) used in the present study was in the same range as it was equivalent to an intake of approximately 732 mg/60 kg human body weight, when calculated on the basis of normalization to the body surface area as described by the recommendations by Reagan-Shaw et al. [24] and the US Food and Drug Administration (http://www.fda.gov/cder/cancer/animalframe.htm). Sibutramine is a monoamine-reuptake inhibitor [25] which was approved as an antiobesity drug by the FDA in 1997 and was marketed until 2010. In the present study, sibutramine was used as a positive control to evaluate the antiobesity effects of OLE.

OLE significantly decreased visceral fat-pad weight and plasma levels of triglyceride and FFA in HFD-fed mice. The decreased visceral fat accumulation might result in the reduced plasma level of FFA. The lower content of FFA, which flows into the liver through the portal vein, may cause less triglyceride synthesis in the liver, resulting in the decreased plasma level of triglyceride in OLD-fed mice. Histological analysis revealed a significantly decreased adipocyte size and an increased number of adipocyte in the OLD group than in the HFD group.

Several olive leaf constituents have been reported to exert beneficial effects against obesity both* in vitro* and *in vivo.* Oleuropein [23, 26], hydroxytyrosol [26, 27], luteolin [28], apigenin [29, 30], rutin [31], and caffeic acid [32] were found to decrease the accumulation of intracellular lipid, as detected by Oil Red O staining, and the expression of adipogenic transcription factors such as PPAR*γ* and C/EBP*α* in 3T3-L1 cells. Hydroxytyrosol also exerts beneficial effects on the promotion of mitochondrial biogenesis via stimulation of the PGC-1*α* and its downstream target genes in 3T3-L1 cells [27]. Oleuropein and rutin decreased body weight gain and improved plasma lipid profiles and hepatic steatosis in HFD-fed mice [23, 31]. Caffeic acid also exhibited antiobesity effects by reducing body and visceral fat-pad weights, plasma levels of lipids, and obesity-related hormones such as leptin and insulin in HFD-fed mice [32]. On the basis of the results of these studies, we speculate that the weight-lowering effects of OLE can be derived from the combination of various weight-suppressing components.

Oleuropein, the bitter principle in olive leaf, accounted for 16% of the OLE used in this study. In our previous research, 0.03% oleuropein supplementation significantly reduced body weight gain (−55%) and plasma lipid levels in HFD-fed mice for 10 weeks [23]. In the present study, OLD (0.15% OLE-supplemented diet) contained 0.024% oleuropein and significantly decreased body weight gain (−80%) and obesity-related plasma biomarkers in mice after 8 weeks of feeding. Although OLD in the present study contained less oleuropein and was fed for a shorter period, it exerted better weight-lowering effects than those exhibited by oleuropein-supplemented diet in our previous study [23]. In addition, OLE significantly reduced food intake in the present study, whereas oleuropein alone did not affect food intake in mice [23]. Therefore, we suggest that the body weight-lowering effect of OLE can be derived not only from oleuropein but also from other active components present in olive leaves that may act concomitantly to reduce food intake and body weight gain.

OLE significantly reduced food intake by 26% in HFD-fed mice for 8 weeks. Several animal studies on OLE investigated its effects on glucose metabolism, ultraviolet B irradiation-induced skin changes, and wound healing [33–35]. Unfortunately, none of the studies presented data for daily food intake. It is well-known that GLP-1 is a satiety-signaling hormone that inhibits food intake by upregulating the expression of anorexigenic neuropeptides such as proopiomelanocortin (POMC) and cocaine- and amphetamine-regulated transcript (CART) [36]. In the present study, the plasma GLP-1 level was not measured because freshly collected plasma samples were not treated with dipeptidyl peptidase IV (DPP IV) inhibitor; without DPP IV inhibitor, GLP-1 is rapidly degraded in plasma. Green et al. demonstrated that oral gavage of dichloromethane extract of olive leaves improved glucose metabolism by significantly increasing plasma GLP-1 concentrations in mice [33]. Among the constituents of OLE, apigenin is known to inhibit food intake at the dosage of 0.05% by increasing the expression of POMC and CART in HFD-fed mice [29]. OLD used in the present study may contain much less apigenin than 0.05%, as, in an earlier report, leaf extract of *Olea europaea* L. contained 1.37% apigenin [37]. Further investigation is needed to confirm whether the food intake-reducing effect of OLE is exerted by its apigenin component.

Food efficiency ratio (FER), which is the ratio of body weight gain (g) to food intake (g), assesses utilization of food consumed. The unchanged FER demonstrates that the weight-lowering effect of a certain antiobesity agent is completely attributable to reduction in food intake. In the present study, OLE significantly decreased FER in HFD-fed mice, indicating that weight-lowering effect of OLE is mediated not only by the reduction of food intake but also by its direct effects on adipocytes. The effect of OLE in reducing the expression of adipogenic genes could be derived from both the direct inhibition of adipogenesis and the reduction of total caloric intake. PPAR*γ* and C/EBP*α* [38] are the chief regulators that act synergistically to induce the expression of downstream target genes of adipogenesis, including FAS, CD36, and leptin. Animal studies have reported that WNT10b [4] and galanin [5] played important roles in regulating the expression of PPAR*γ*2 and C/EBP*α*. WNTs, secreted proteins that act through autocrine and paracrine mechanisms, serve as a functional “brake” during preadipocyte recruitment into the differentiation program [3]. Of the 19 possible WNTs, WNT10b has been most clearly implicated as the endogenous WNT involved in regulating adipogenesis [39]. HFD decreased the binding of WNT10b to FZD receptors and to LRP5/6 coreceptors, leading to the activation of the cytoplasmic *β*-catenin degradation [6]. The decreased cytosolic accumulation and nuclear translocation of *β*-catenin led to decreased transcriptional coactivation of the T-cell factor (TCF)/lymphoid-enhancer factor (LEF) transcription factors, and eventually, upregulated the expression of PPAR*γ* and C/EBP*α* [40]. OLE significantly reversed the HFD-induced decrease in the expression of WNT10b and LRP5, which may be associated with increased cytosolic accumulation of *β*-catenin.

In addition, it is noteworthy that the WNT signaling pathway is regulated by endogenous WNT inhibitors [3]. SFRP5, a WNT inhibitor, directly binds to and sequesters WNT10b from FZD receptors. Epigenetic activation of SFRP5 in WAT increased susceptibility to obesity in HFD-fed mice [41]. In the present study, SFRP5 expression was significantly reduced in the epididymal adipose tissue of OLD-fed mice than in HFD-fed mice. Therefore, we presume that OLE-mediated downregulation of WNT10b inhibitor, SFRP5, and upregulation of the ligand, WNT10b, may be related to the increased cytosolic accumulation of *β*-catenin and thus the reduction in adipogenesis and visceral adiposity.

The galanin-mediated signaling pathway is also involved in the regulation of adipogenesis. Galanin, a 29/30-residue neuropeptide, is expressed and widely distributed in the central nervous system [42]. However, recent studies have revealed that galanin expression has also been observed in peripheral tissues such as visceral adipose tissue and stomach [5]. Our previous study demonstrated that the expression of galanin, its receptors, and other genes involved in galanin-mediated signaling pathway was significantly elevated in the epididymal adipose tissues of HFD-fed mice [5]. Upon prolonged HFD consumption, GalR1 stimulated the mitogen-activated protein kinase activity in a PKC-independent manner and activated 1/2 ERKs. Activation of GalR2 increased the expression of phospholipase C (PLC) which catalyzes the cleavage of phosphatidylinositol 4,5-bisphosphate into inositol 1,4,5-triphosphate and diacyl glycerol, a PKC activator. Subsequently, PKC*δ* induced the activation of ERK, which upregulated the expression of PPAR*γ* and C/EBP*α*. In the present study, OLE significantly reversed HFD-induced elevation of the expression of genes (GalR1, GalR2, PKC*δ*, and ERK) in the epididymal adipose tissue of mice. Therefore, the effect of OLE on the reduction of visceral fat-pad weights is presumed to be associated with the downregulation of genes related to galanin-mediated adipogenesis in HFD-fed mice.

In addition to the inhibition of fat accumulation in WAT, improvement of the capacity of thermogenesis can also account for the body fat-lowering effect of OLE. Adaptive thermogenesis is defined as heat production in response to environmental temperature or diet, which protects the organism from exposure to cold or regulates the energy balance after changes in the diet [1]. Mitochondria, the organelles that convert food to carbon dioxide, water, and ATP, are indispensable for energy dissipation. Activation of UCP1, a mitochondrial proton transporter, enhances uncoupled respiration and thereby results in dissipation of oxidation energy as heat [1]. Galanin receptors [5] and SIRT1 [43] are known to reverse obesity-induced downregulation of UCP1 expression in WAT. Our previous study demonstrated that the HFD-induced overexpression of GalR1 and GalR2 inhibited cAMP, which in turn reduced protein kinase A (PKA), in the epididymal and subcutaneous adipose tissues of mice. Decreased PKA downregulated the phosphorylation of CREB, leading to decreased gene expression of PGC-1*α* [5]. SIRT1, which is responsible for deacetylating and activating PGC-1*α* at multiple lysine sites, was downregulated by HFD in the adipose tissue of mice [44]. Consequently, HFD-induced downregulation of PGC-1*α* decreased the expression of UCP1 and eventually suppressed uncoupled respiration. In the present study, OLE simultaneously reversed the HFD-induced upregulation of GalR1 and GalR2 expression and downregulation of SIRT1, PGC-1*α*, and UCP1 expression in the epididymal adipose tissue of mice. Therefore, we suggest that the visceral fat-pad weight-lowering effects of OLE can be associated with the elevation of the gene expression involved in uncoupled respiration in the visceral adipose tissue of HFD-fed mice.

The coordination of mitochondrial biogenesis with uncoupled respiration is crucial in increasing the capacity of thermogenesis [1]. PGC-1*α* is a potent inducer of mitochondrial biogenesis. Shadel and Clayton demonstrated that PGC-1*α* led to an activation of nuclear respiratory factor-1 (NRF-1), which binds to and activates the gene promoter of the mitochondrial electron transport system such as COX2. Another target of NRF-1 is TFAM, which translocates into mitochondria and initiates the transcription and replication of the mitochondrial DNA [45]. OLE significantly increased the mRNA expression of genes involved in mitochondrial biogenesis (PGC-1*α*, NRF-1, COX2, and TFAM) in the epididymal adipose tissue of HFD-fed mice. Accordingly, we presume that the visceral adiposity-suppressing effects of OLE may be linked to the increased mitochondrial biogenesis-related genes in the epididymal adipose tissue of mice.

In conclusion, OLE significantly decreased body weight gain, visceral fat-pad weights, and plasma lipid levels in HFD-fed mice. These beneficial effects against obesity in mice appear to be mediated, at least in part, through downregulating the expression of molecules involved in adipogenesis and upregulating the expression of molecules involved in thermogenesis in the visceral adipose tissue of HFD-fed mice. Taken together, our results suggest that supplementation with OLE might be helpful to combat or prevent obesity.

## Figures and Tables

**Table 1:** Composition of the experimental diets.

Ingredients	HFD (g/kg diet)	OLD (g/kg diet)	SD (g/kg diet)
Casein	200	200	200
DL-methionine	3	3	3
Corn starch	111	109.5	110.9
Sucrose	370	370	370
Cellulose	50	50	50
Corn oil	30	30	30
Lard	170	170	170
Mineral mixture^1^	42	42	42
Vitamin mixture^2^	12	12	12
Choline bitartrate	2	2	2
Cholesterol	10	10	10
*tert*-Butylhydroquinone^3^	0.04	0.04	0.04
Olive leaf extract	—	1.5	—
Sibutramine	—	—	0.1

Total (g)	1,000	1,000	1,000
Fat (% calories)	40	40	40
Total energy (kcal/kg diet)	4,616	4,616	4,616

^1^AIN-76A mineral mix.

^2^AIN-76A vitamin mix.

^3^Antioxidant agent: 0.01 g/50 g lipids.

**Table 2:** Primer sequences and RT-PCR conditions.

Gene description	Primers	Sequences (5′→3′)	*T*_*m*_ (°C)	Size (bp)
Peroxisome proliferator-activated receptor *γ* (PPAR*γ*)	F	TTCGGAATCAGCTCTGTGGA	55	148
	R	CCATTGGGTCAGCTCTTGTG		
CCAAT/enhancer binding-protein *α* (C/EBP*α*)	F	AAGGCCAAGAAGTCGGTGGA	55	189
	R	CCATAGTGGAAGCCTGATGC		
Leptin	F	CTCCAAGGTTGTCCAGGGTT	55	143
	R	AAAACTCCCCACAGAATGGG		
Cluster of differentiation 36 (CD36)	F	ATGACGTGGCAAAGAACAGC	55	160
	R	GAAGGCTCAAAGATGCCTCC		
Fatty acid synthase (FAS)	F	TTGCCCGAGTCAGAGAACC	55	171
	R	CGTCCACAATAGCTTCATAGC		
Wingless-type MMTV integration site family, member 10b (WNT10b)	F	TTTTGGCCACTCCTCTTCCT	61	183
	R	TCCTTTTCCAACCGAAAACC		
Secreted frizzled-related protein 5 (SFRP5)	F	CTGATGGCCTCATGGAACAG	55	155
	R	CTTGGTGTCCTTGCGCTTTA		
Galanin	F	GAGCCTTGATCCTGCACTGA	60	121
	R	AGTGGCTGACAGGGTCACAA		
Galanin receptor 1 (GalR1)	F	CCAAGGGGGTATCCCAGTAA	55	147
	R	GGCCAAACACTACCAGCGTA		
Galanin receptor 2 (GalR2)	F	ATAGTGGTGCTCATGCTGGAA	60	134
	R	AGGCTGGATCGAGGGTTCTA		
Protein kinase C *δ* (PKC*δ*)	F	CTGAGCGCTGCAAGAAGAAC	60	146
	R	TGGAAACTTTGATCCTGCACTGA		
Peroxisome proliferative activated receptor *γ* coactivator 1*α* (PGC-1*α*)	F	TAAATCTGCGGGATGATGGA	55	117
	R	GTTTCGTTCGACCTGCGTAA		
Uncoupling protein 1 (UCP1)	F	GGTTTTGCACCACACTCCTG	55	111
	R	ACATGGACATCGCACAGCTT		
Cyclin D (cyc-D)	F	TGGGAAGTTTTGTTGGGTCA	55	144
	R	TCCTTGTCCAGGTAATGCCA		
E2F1	F	CCTGGAGCATGTTAAAGAAG	55	102
	R	CCTCGAGACCAAAGTGATAG		
Sirtuin 1 (SIRT1)	F	AGCTCCTTGGAGACTGCGAT	55	182
	R	ATGAAGAGGTGTTGGTGGCA		
Cytochrome C oxidase subunit 2 (COX2)	F	CCGAGTCGTTCTGCCAATAG	55	159
	R	AACCCTGGTCGGTTTGATGT		
Mitochondrial transcription factor A (TFAM)	F	AGTGTGGCAGTCCATAGGCA	55	123
	R	CAGTGCTTTTAGCACGCTCC		
Nuclear respiratory factor-1 (NRF-1)	F	TTTCATGGACCCAGGCATTA	55	119
	R	TGGTGGCCTGAGTTTGTGTT		
Glyceraldehyde-3-phosphate dehydrogenase (GAPDH)	F	AGAACATCATCCCTGCATCC	55	321
	R	TCCACCACCCTGTTGCTGTA		

## Abbreviations

C/EBP*α*: CCAAT/enhancer-binding protein alpha
CD: Chow diet
CD36: Cluster of differentiation 36
CREB: cAMP responsive element-binding protein
Cyc-D: Cyclin D
COX2: Cytochrome C oxidase subunit 2
ERK: Extracellular signal-regulated kinase 1/2
FAS: Fatty acid synthase
FFA: Free fatty acids
GalR1: Galanin receptor 1
GalR2: Galanin receptor 2
GAPDH: Glyceraldehyde-3-phosphate dehydrogenase
HFD: High-fat diet
NRF-1: Nuclear respiratory factor-1
OLE: Olive leaf extract
PGC1*α*: Peroxisome proliferator-activated receptor gamma coactivator 1alpha
PPAR*γ*: Peroxisome proliferator-activated receptor gamma
PKC*δ*: Protein kinase C delta
SFRP5: Secreted frizzled-related protein 5
SIRT1: Sirtuin 1
TFAM: Mitochondrial transcription factor A
UCP1: Uncoupling protein 1
WNT10b: Wingless-type MMTV integration site family, member 10b.
